# 

**Published:** 1890-04-05

**Authors:** 


SATURDAY, APRIL 5th, 1890.

**Country Roads** - 1

**Words of Consolation.**—The Lord is Risen - 2

**The Provident Principle in Connection with Hospitals.** By Sir Edmund Hay Currie - 3

**A Hint from America** - 4

**The Doctor's Pulpit.**—Women's Uncongenial Occupations - 5

**The History and Capabilities of Herbal Simples.** By W. T. Fernie, M.D. VII. Wood Betony - 6

**Everybody's Page** - 7

**Notes and News** - 8

**Annotations.**—Politicians and Physicians—Canal Boats and their Occupants—The Fasting Man - 10

**Scraps and Gleanings** - 16

**The Practitioner's Mirror and Retrospect:**

Medicine—Hysteria and Organic Disease—Gastric Catarrh - 11

Surgery—Colotomy—Renal Calculus - 12

New Drugs, Appliances, and Things Medical.—The Horton Ice-Cream Company—Santha—Godfrey's Chloride of Ammonium Inhaler - 13

Hygiene—The Bacillus of Typhoid - 13

Therapeutics—Calomel for Diphtheria - 14

The Practitioner's Bookshelf.—The Medical Annual - 14

**The Hospital Workshop**—XXIV. The Children's Hospital, Birmingham - 15

**Passing Topics.**—The C.O.S. and the Preparation of Charitable Accounts - 16

EXTRA SUPPLEMENT—THE NURSING MIRROR.

**En Passant.**—Bolton Nursing Association—Lincoln Institution—Loyalty—Short Items—Thousands of Pounds for the National Pension Fund—District Nursing at Staleybridge — Exhibition of Nursing Uniforms—Nursing in Chicago - i

**Nursing Lectures.** By Percy Lewis, M.D. VII.—Abdominal Diseases - ii

**Presentations** - ii

**News from Tasmania** - iii

**Everybody's Opinion.**—Meum and Tuum—A Protest from the Ranks—Nurses' Mutual Society - iii

**Examination Questions—Appointments** - iv

**Notes and Queries—Vacancies** - iv

**Amusements and Relaxation** - iv

